# Examining the Role of Information Behavior in Linking Cancer Risk Perception and Cancer Worry to Cancer Fatalism in China: Cross-Sectional Survey Study

**DOI:** 10.2196/49383

**Published:** 2024-05-31

**Authors:** Lianshan Zhang, Shaohai Jiang

**Affiliations:** 1 School of Media and Communication Shanghai Jiao Tong University Shanghai China; 2 Department of Communications and New Media National University of Singapore Singapore Singapore

**Keywords:** cancer fatalism, cancer risk perception, cancer worry, health information seeking, information avoidance

## Abstract

**Background:**

Reducing cancer fatalism is essential because of its detrimental impact on cancer-related preventive behaviors. However, little is known about factors influencing individuals’ cancer fatalism in China.

**Objective:**

With a general basis of the extended parallel process model, this study aims to examine how distinct cancer-related mental conditions (risk perception and worry) and different information behaviors (information seeking vs avoidance) become associated with cancer fatalism, with an additional assessment of the moderating effect of information usefulness.

**Methods:**

Data were drawn from the Health Information National Trends Survey in China, which was conducted in 2017 (N=2358). Structural equation modeling and bootstrapping methods were performed to test a moderated mediation model and hypothesized relationships.

**Results:**

The results showed that cancer risk perception and cancer worry were positively associated with online health information seeking. In addition, cancer worry was positively related to cancer information avoidance. Moreover, online health information seeking was found to reduce cancer fatalism, while cancer information avoidance was positively associated with cancer fatalism. The results also indicated that the perceived usefulness of cancer information moderated this dual-mediation pathway.

**Conclusions:**

The national survey data indicate that cancer mental conditions should not be treated as homogeneous entities, given their varying functions and effects. Apart from disseminating useful cancer information to encourage individuals to adaptively cope with cancer threats, we advocate for health communication programs to reduce cancer information avoidance to alleviate fatalistic beliefs about cancer prevention.

## Introduction

### Background

Cancer is rapidly becoming a global health burden and is the leading cause of death in >110 countries [1]. In China, the context for this study, cancer incidence and mortality have escalated, with an estimated 4.8 million new cancer cases and 3.2 million new cancer deaths in 2022, approximately 40% higher cancer mortality than in the United States [2]. Despite the crude cancer deaths, studies have found that globally 40% to 50% of cancers are preventable by choosing positive lifestyle factors, such as following a healthy diet, maintaining regular exercise and cancer screenings, and reducing tobacco use and alcohol consumption [3].

To promote cancer prevention, fostering positive coping beliefs is an essential step. However, many people still hold fatalistic beliefs about cancer, considering it as neither preventable nor curable [4]. Those with fatalism contend that external forces, such as fate and predestination, control the causes and outcomes of cancer and hence deny the need to engage in any other form of coping [5]. Such maladaptive coping modes have been documented in both Eastern and Western societies, despite limited studies in Asia [6]. In China, cancer fatalism has long been prevalent, and it carries a negative connotation (eg, hopelessness and pessimism) that is associated with negative action tendencies in the face of cancer risks [7].

Hence, it is important to reduce cancer fatalism, and health information seeking may play a key role. Past research has documented the benefits of health information seeking, such as lowering health anxiety, managing health-related uncertainties, and increasing health literacy and confidence in fighting cancer [8]. However, people are not always active in searching for health information. Instead, some people intentionally avoid cancer information or prevent exposure to related topics, which is called cancer information avoidance (CIA) [9]. People who consistently avoid cancer information may miss opportunities to be empowered in making informed decisions to take positive coping behaviors. In past studies, CIA has shown to be associated with low levels of perceived behavioral control, cancer knowledge, and delays in seeking help [10,11]. Although the detrimental impacts of CIA on maladaptive coping have been suggested, prior research predominantly concentrated on health information seeking, inadvertently overlooking the simultaneous examination of information seeking and CIA as distinct appraisals within the context of cancer fatalism development from the theoretical lens of the extended parallel process model (EPPM). This narrow focus hinders a comprehensive understanding of various information behaviors and their potentially varying implications on fatalistic beliefs concerning cancer prevention, particularly considering that CIA is more prevalent than avoidance of any other disease-related information given its threatening nature [12]. Thus, it is valuable to investigate how cancer fatalism may be influenced by 2 distinct appraisals—the danger control process related to information seeking and the fear control process related to CIA—which are believed to have apparently contrasting effects on cancer fatalism.

To investigate why some people actively seek cancer information on their own initiative while others choose to avoid it, one must take into account cancer-related affect and cognition, such as cancer worry and cancer risk perception [13]. Noticeably, cancer worry and cancer risk perception are distinct constructs, and they act in different ways in influencing people’s information behavior [14]. However, how distinct cancer mental conditions are associated with different information behaviors (seeking vs avoidance), which further become associated with cancer fatalism, has not been addressed. As mentioned earlier, the EPPM provides a guiding theoretical framework for our examination, which demonstrates that in the face of a threat, individuals may engage in different information responses (adaptive vs maladaptive) based on their risk appraisals, which can further make a difference in outcome variables such as one’s threat coping tendencies [15]. Considering that cancer fatalism involves individuals who deny their coping or behavioral needs [16-18], it is reasonable to expect that different information behaviors that individuals engaged in would be associated with different levels of fatalism, reflecting individuals’ negative coping needs.

Apart from cancer-related mental conditions, people’s information behavior is also influenced by information-carrier characteristics, for example, perceived cancer information usefulness, especially in the complex digital information environment [19]. In our study context, individuals would perceive the information to be useful if they deem the information can provide them with useful information or resources to deal with cancers. In this sense, perceived cancer information usefulness can be understood as a manifestation of response efficacy (eg, a belief as to whether a recommended response works in preventing a given threat) from the theoretical perspective of EPPM. Moreover, EPPM articulates that whether individuals engage in adaptive response (eg, information seeking) or maladaptive response (eg, information avoidance) depends on the interplay between threat appraisals (eg, severity and susceptibility) and efficacy appraisals (eg, response efficacy), suggesting the moderating role cancer information usefulness plays in our study.

The EPPM has traditionally been applied to elucidate how perceived self-threat influences an individual’s coping tendencies in the context of explicit message persuasion. However, a growing body of research has expanded the EPPM’s scope to include contexts beyond message persuasion, such as the incidental influence context [20]. While originally not designed for persuasion purposes, media coverage of health concerns has been found to incidentally influence variables relevant to public health, such as risk perceptions and effective responses [21,22]. This is not surprising, given that individuals need to be made aware of potential threats, and authorities are tasked with providing guidance on how to address them [23]. Consequently, the EPPM has been a valuable framework for application in nonpersuasion contexts to understand why and how people respond to health threats, often influenced by daily exposure to media reports containing persuasive health messages [23]. Hence, building upon the core tenets of the EPPM and drawing from prior empirical studies applying the EPPM to nonpersuasion contexts [24,25], one of the objectives of our study is to examine how individuals’ subjective evaluation of a threat (ie, cancer) becomes associated with their coping responses via 2 appraisals in a nonpersuasion context. Within this context, the perceived threat is expected to be shaped by persuasive health messages that individuals encounter daily in the media. In this regard, it is important to note that our study does not seek to examine the effects of the intentionally crafted persuasive message on health outcomes (eg, attitude or behavioral change) or to test all the postulations of the EPPM. Instead, our focus centers on predicting individuals’ coping responses through 2 appraisals (ie, danger control and fear control), which are grounded in their subjective evaluations of a threat and efficacy.

**Figure 1:**
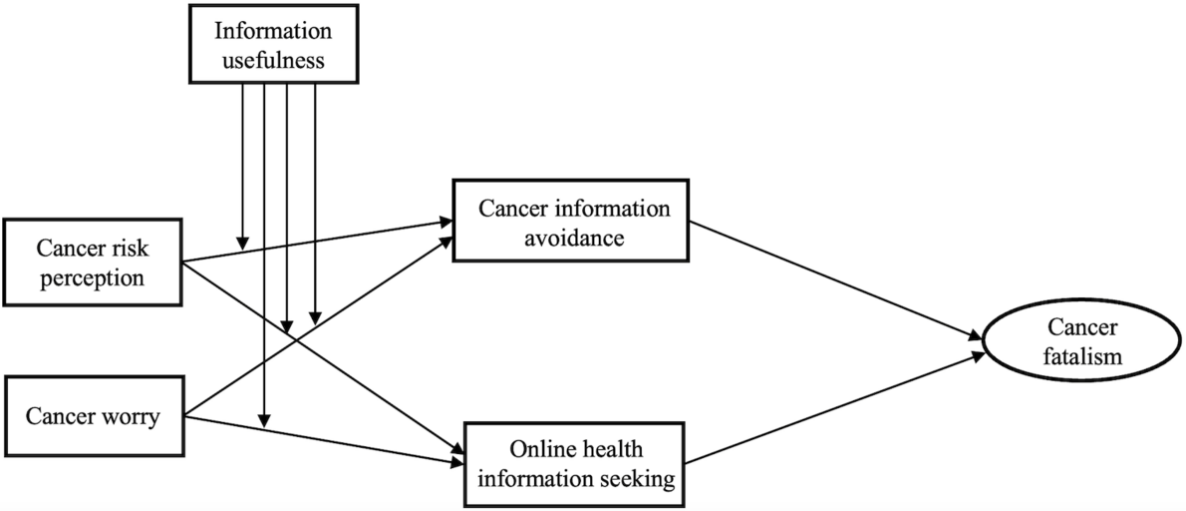
Hypothesized model.

In light of the above, this study examines the path from 2 distinct cancer mental conditions (cancer worry and cancer risk perception) to 2 information behaviors (health information seeking and CIA) and further onward to cancer fatalism, considering the moderating role of perceived cancer information usefulness (Figure 1). The next sections discuss the key concepts of this study and offer evidence for the proposed pathways.

### Study Hypotheses

#### Cancer Risk Perception, Cancer Worry, and Information Behavior

Cancer risk perception and cancer worry are salient cancer-related thoughts and feelings that have been frequently investigated. Specifically, cancer risk perception has been largely conceptualized as one’s cognitive evaluation of perceived susceptibility to getting cancer, whereas cancer worry has been primarily regarded as an affective response to cancer threat [26,27]. In particular, Chae [14] developed a cancer-related mental condition model that differentiated cancer worry and cancer risk perception. She contended that cancer worry is a more affective condition compared to cancer risk perception, a more cognitive state. In other words, cancer worry is a mental activity that is closely linked to one’s emotions (eg, anxiety and fear) triggered by cancer threats and thus an affective-cognitive condition. Cancer risk perception centers on one’s rational judgment of the likelihood of getting cancer, which often involves deliberative and intellectual assessment and thus a cognitive appraisal.

Previous studies have documented ample evidence in linking cancer risk perception and cancer worry to health information seeking. For example, Nan et al [28] found that higher levels of cancer risk perception were associated with a greater likelihood of seeking prostate and breast cancer information. Yoo et al [29] indicated that people who perceived themselves with a high degree of getting cervical cancer were more likely to seek health information on social media. In the same vein, heightened cancer worry has been argued as a motivator for information acquisition. For instance, Griffin et al [30] demonstrated that personal worry prompts one’s information needs in coping with health risks. The planned risk information seeking model [31] and its subsequent studies further confirmed the positive association between personal worry and searches for health information. Consistent with previous research, this study has the following hypotheses:

- Hypothesis 1: Cancer risk perception will be positively associated with online health information seeking (OHIS).
- Hypothesis 2: Cancer worry will be positively associated with OHIS.

Despite such motivational triggers, a growing body of research has made a seemingly competing argument, stating that cancer risk perception and cancer worry may lead to more CIA. For instance, Moser et al [32] found that cancer is a substantial threat to many people who consider it a death sentence, increasing their fear and anxiety. Under such circumstances, people refuse to be exposed to cancer-related information to reduce uncomfortable feelings [33]. This inhibiting role of cancer worry and risk perception is also elucidated by the EPPM [15], which demonstrates 2 appraisals people may adopt in dealing with threats. On the one hand, when people perceive a high appraisal of threat (eg, heightened risk perception), they may be activated to take adaptive actions (eg, information seeking) to better equip themselves in coping with the threat, known as the danger control process. On the other hand, people might choose defensive avoidance (eg, information avoidance) to escape the potential of eliciting negative emotions and feelings, known as the fear control process. In line with this notion of the EPPM, some people would engage in CIA in reducing unconformable feelings, especially when they perceive a high degree of cancer threats [34]. Several studies provide empirical evidence for this argument [11]. For example, Case et al [35] demonstrated that people tended to avoid or ignore threatening information to manage emotional states such as anxiety and fear. Vrinten et al [11] also found that CIA significantly increased, as cancer worry escalated. Hence, in light of prior literature, this study postulates the following hypotheses:

- Hypothesis 3: Cancer risk perception will be positively associated with CIA.
- Hypothesis 4: Cancer worry will be positively associated with CIA.

#### OHIS, CIA, and Fatalistic Beliefs About Cancer Prevention

Fatalism, a deterministic outlook that one’s health is controlled by external forces, and therefore, there is no need to engage in positive coping behaviors, has been viewed as a prominent barrier to cancer prevention and screening behaviors [5,36,37]. By definition [5], cancer fatalism can be understood as one’s negative behavioral tendency (eg, no need to cope and refusing coping behaviors) in the face of cancer threats [16,18]. Although some studies have approached cancer fatalism as a concept embedded in culture, primarily investigating its influence on information behaviors [12,38], we argue that cancer fatalism is a malleable concept that can be intervened through media learning and health education, such as information and knowledge acquisition from media use. In fact, numerous empirical studies have provided strong evidence of the positive impact of educational attainment and health literacy in reducing cancer fatalism [10,39-42]. These findings suggest that diverse information behaviors (ie, seeking and avoidance), involving varying levels of media exposure and educational opportunities, can make a significant difference in shaping the development of cancer fatalism. Thus, it is both reasonable and essential to examine the relationship from information behaviors to cancer fatalism. It is worth noting that both cancer-specific information seeking and general health information seeking are beneficial. While cancer-specific information seeking aids in gaining cancer-related knowledge, general health information seeking is effective in narrowing disparities in health literacy, thereby reducing cancer fatalism [43]. Particularly in this digital era, the internet offers convenient access to health information. With useful health information, patients have a better understanding of their health conditions, prescription drugs, treatments, and disease management options, which can empower them, reducing cancer fatalism [41,44]. Health information exchange with physicians or peers on the internet may also encourage individuals to take a more active role in preventive behaviors, lowering fatalistic beliefs about cancer [43]. By contrast, if people intentionally avoid cancer information, they might lose opportunities to receive information relevant to them, increasing health uncertainties and cancer fatalism [33].

Our reasoning is well aligned with the theoretical standpoint of EPPM, which demonstrates that the 2 information responses (adaptive vs maladaptive) that individuals adopt driven by their threat appraisals would lead to disparities in outcome variables such as one’s threat coping tendency. Contextualized in this study, individuals who take adaptive actions in engaging in health information seeking tend to be well equipped with cancer-related knowledge, which in turn helps eliminate their fatalistic belief about cancer prevention, whereas individuals who choose defensive steps in engaging in information avoidance are more likely to be vulnerable to cancer fatalism due to their refusing coping behaviors [18]. A couple of empirical studies have also illustrated that CIA can lead to fatalistic beliefs about cancer and less frequent cancer screenings [10,17]. Hence, based on prior literature, we proposed the following hypotheses:

- Hypothesis 5: OHIS is negatively associated with fatalistic beliefs about cancer prevention.
- Hypothesis 6: CIA is positively associated with fatalistic beliefs about cancer prevention.

So far, this study reviewed 2 well-established relationships that link 3 elements: cancer mental conditions, information behaviors, and cancer fatalism. Given the established 2-step relationship, an underlying dual pathway between cancer risk perception and cancer fatalism as well as between cancer worry and cancer fatalism is likely to be mediated by OHIS and CIA, which suggests the following hypotheses:

- Hypothesis 7: OHIS will mediate (1) the relationship between cancer risk perception and cancer fatalism and (2) the relationship between cancer worry and cancer fatalism.
- Hypothesis 8: CIA will mediate (1) the relationship between cancer risk perception and cancer fatalism and (2) the relationship between cancer worry and cancer fatalism.

#### Moderating Role of Perceived Usefulness of Online Cancer Information

Given the dynamic process of information seeking that involves interactions between information seekers and information platforms, we need to consider how information seekers perceive health information. Specifically, we investigated the moderating role of one’s perceived information usefulness, a vital information-carrier predictor of individuals’ information behavior [45]. Barbour et al [46] demonstrated that if people viewed health information as questionable and unclear, they tended to avoid such information to reduce stress and uncertainties despite their serious illnesses. A review study also concluded that the decision to seek or avoid cancer information was contingent upon situational factors, such as the usefulness of the information [33]. As Johnson [45] posited, information seekers are concerned about the content of the information. They put greater effort into seeking information that is deemed useful in coping with their cancer threats. Conversely, if they consider the information to be less effective, they may have a higher tendency to avoid it.

Moreover, echoing the EPPM [15], engaging in fear (information avoidance) or danger control (information seeking) is a synergistic effect of 2 appraisals: threat (eg, severity and susceptibility) and efficacy (eg, response efficacy and self-efficacy). Specifically, response efficacy refers to the perception of whether the provided information or recommended response works in allaying the threat [34]. Particularly relevant to the information environment, useful cancer information is a typical resource that offers people informational and emotional strategies to cope with threats [47]. Therefore, conceptualizing the usefulness of cancer information as a manifestation of response efficacy, it is expected that the relationship between one’s cancer-related mental conditions and information behavior will be moderated by the perceived usefulness of cancer information from the theoretical perspective of EPPM. Accordingly, the following hypothesis is posited:

- Hypothesis 9: The perceived usefulness of online cancer information will moderate (1) the relationship between cancer risk perception and OHIS, (2) the relationship between cancer risk perception and CIA, (3) the relationship between cancer worry and OHIS, and (4) the relationship between cancer worry and CIA.

## Methods

### Data and Participants

This study used cross-sectional data from the Health Information National Trends Survey (HINTS) in China (HINTS-China). Similar to the HINTS that has been implemented in the United States since 2003, the current HINTS-China was conducted in May 2017. HINTS-China is an international collaboration involving the National Cancer Institute, the Chinese Ministry of Health, and the Chinese Food and Drug Administration, in conjunction with George Mason University. It was initially established with Renmin University of China and has subsequently collaborated with Beijing Normal University [48]. A multistage stratified random sampling method was adopted, and a door-to-door survey method was used. Specifically, 2 cites were purposely chosen due to their representativeness: Beijing (representing a tier-1 city) and Hefei (representing a tier-2 city). Then, 1 urban district (representing an urban area) and 1 rural county (representing a rural area) were randomly selected in each of the 2 cities. Within each urban district and rural county, 1 subdistrict and township was randomly selected from 3 strata by the level of economic development (high, medium, and low). A total of 4 residential neighborhoods were then randomly selected from each subdistrict and township stratified by sex and age (for detailed sampling methodology, refer to the study by Zhao et al [49]).

A total of 3090 respondents completed the survey. In this study, we only included those who had internet access, as 1 focal variable was OHIS. In addition, patients with cancer were excluded from our sample because 1 key variable, cancer risk perception, measured people’s evaluation of the likelihood of getting cancer. Therefore, the final sample size in this study was 2358. The participants’ mean age was 33.98 (SD 10.88, range 18 to 60) years. In total, 60.3% (1421/2358) were female. More than half of the participants (1332/2358, 56.49%) obtained some college education or more. Less than a third (705/2358, 29.9%) earned monthly income >CNY 5000 (US$700). Approximately 94.44% (2227/2358) of the respondents had health insurance coverage, and 16% (377/2358) had at least 1 chronic condition. The average self-reported health condition was at the “good” level (mean 3.98, SD 0.76).

### Ethical Considerations

The HINTS-China was approved by the institutional review board (IRB) at Beijing Normal University in 2017. Respondents who participated in the survey gave their written consent. The data were deidentified and publicly available [50]. Secondary data analysis using the HINTS-China data set in our study did not need to obtain IRB approval because research involving the study of existing data, if these sources are publicly available or research participants cannot be identified, is in the exemption category of IRB [51]. This is also a common practice in prior research using HINTS-China data [50,52].

### Measures

#### Cancer Risk Perception

Drawing from prior research examining cancer risk perception [14,53], respondents were asked to indicate their judgment of the likelihood of getting cancer on a 5-point Likert scale (1=*very unlikely* and 5=*very likely*): “Compared to the average person of your age and sex, how likely would you rate your chance of developing cancer sometime in your life?” (mean 2.32, SD 0.84).

#### Cancer Worry

Similar to prior studies using HINTS data [54], this study used a single item to ask participants to indicate to what extent they worried about getting cancer on a 5-point Likert scale (1=*not at all* and 5=*extremely*): “How worried are you about getting cancer?” (mean 2.25, SD 1.00).

#### Perceived Usefulness of Cancer Information

Adapted from prior research that used a single item by using HINTS data [55], in examining to what extent respondents considered online cancer information to be useful, participants were asked to rate the overall usefulness of online cancer information on a 4-point Likert scale (1=*not at all useful* and 4=*very useful*; mean 2.35, SD 0.68).

#### OHIS Measure

To investigate the extent to which respondents sought general health information on the internet, we used six items, drawn from previous research [43], that asked participants whether they have carried out the following activities on the internet in the last 12 months (1=*yes* and 0=*no*): (1) looked for a health care provider or information about hospitals, (2) looked for exercise, weight control, or fitness information, (3) looked for information about quitting smoking, (4) looked for health or medical information for someone else, (5) asked and exchanged health-related information and topics, and (6) downloaded health or medical information. A summative scale of these 6 dichotomous items was created (mean 1.03, SD 0.99).

#### CIA Measure

To estimate the extent to which people intentionally avoid cancer information, participants were asked to report their agreement with the following statement on a 5-point scale (1=*strongly disagree* and 5=*strongly agree*) adapted from prior research [9]: “I avoid being exposed to cancer information” (mean 2.76, SD 0.98).

#### Fatalistic Beliefs About Cancer Prevention

Drawing from previous studies using HINTS data [37,41], participants were asked to evaluate their agreement with 3 statements about fatalistic beliefs concerning cancer prevention on a 5-point Likert scale (1=*strongly disagree* and 5=*strongly agree*): (1) “There is not much I can do to lower my chances of getting cancer,” (2) “It seems that everything causes cancer,” and (3) “When I think about cancer, I automatically think about death.” (mean 3.16, SD 0.74; Cronbach α=0.74).

Control variables included social demographics such as age, sex, education, and personal monthly income. In addition, as this study investigated people’s cancer-related beliefs, health-related variables were also controlled, including participants’ general health status (1=*very poor* and 5=*very good*), chronic disease conditions (1=*yes* and 0=*no*), health insurance coverage (1=*yes* and 0=*no*), and family cancer history (1=*yes* and 0=*no*).

### Analytic Approach

To examine the hypothesized model, structural equation modeling was conducted using the lavaan package in R. Maximum likelihood of estimation was adopted. Following the widely used combinational rules and prior research [56], the goodness of fit of the hypothesized model should be (1) Tucker-Lewis index or comparative fit index≥0.95 and standardized root mean square residual (SRMR) ≤0.08, or, alternatively, (2) root mean square error of approximation<0.05 and SRMR <0.06. To assess the mediating effect (ie, hypothesis 7 and hypothesis 8), the bias-corrected bootstrapping method was used to estimate the path CI [57]. A CI that does not include 0 indicates a statistically significant mediating effect at the 95% CI. To examine the moderating effect of the perceived usefulness of cancer information (ie, hypothesis 9), interaction terms between independent variables (ie, cancer risk perception and cancer worry) and the perceived usefulness of cancer information were created, and the 3 variables were centered before forming the interaction terms to reduce multicollinearity problem.

## Results

### Structural Model and Path Coefficients

**Figure 2:**
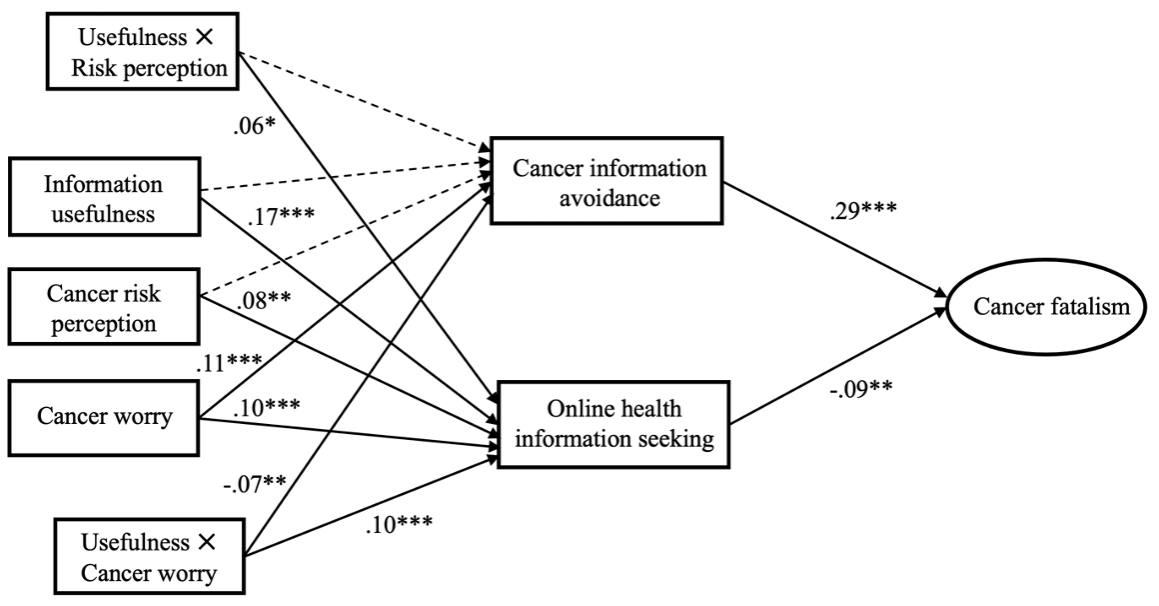
Final model with standardized path coefficients. Dotted lines indicate nonsignificant relationships. The covariances between all exogenous factors (eg, controls) and coefficients with control variables are not presented for the purpose of clarity. *P<.05, **P<.01, ***P<.001.

Table 1 shows the descriptive statistics and bivariate correlations for measured variables. Controlling for social demographics and health-related variables, the structural model showed an acceptable fit (*χ*^2^_92_=254.4; *P*<.001; comparative fit index=0.95; Tucker-Lewis index=0.94; root mean square error of approximation=0.05; 90% CI 0.041 to 0.053; SRMR=0.04). As shown in Figure 2, our findings revealed that cancer risk perception positively predicted OHIS (β=.08; *P*=.007). Similarly, cancer worry was positively related to OHIS (β=.10; *P*<.001), supporting hypothesis 1 and hypothesis 2. In addition, cancer worry was positively associated with CIA (β=.11; *P*<.001), whereas the results indicated a nonsignificant relationship between cancer risk perception and CIA (β=–.03; *P*=.23). Hence, hypothesis 4 was supported but not hypothesis 3. Moreover, the results showed that CIA was positively associated with fatalistic beliefs about cancer prevention (β=.29; *P*<.001); conversely, OHIS was negatively related to cancer fatalism (β=–.09; *P*=.003), supporting hypothesis 5 and hypothesis 6. In total, our hypothesized model explained 25.1% of the variance of cancer fatalism among the participants.

**Table 1:** Descriptive statistics and correlation analysis (Person r and 2-tailed *P* value) among measured variables (N=2358).

Variable		Cancer risk perception		Cancer worry	Perceived usefulness of online cancer information	Online health information seeking	Cancer information avoidance	Fatalistic beliefs about cancer prevention
**Cancer risk perception** **(mean 2.32, SD 0.84)**								
	*r*	1	0.51		0.10	0.15	0.05	0.12
	*P* value	—^a^	<.001		<.001	<.001	.01	<.001
**Cancer worry** **(mean 2.25, SD 1.00)**								
	*r*	0.51	1		0.12	0.16	0.11	0.15
	*P* value	<.001	—		<.001	<.001	<.001	<.001
**Perceived usefulness of online cancer information** **(mean 2.35, SD 0.68)**								
	*r*	0.10	0.12		1	0.18	0.03	0.15
	*P* value	<.001	<.001		—	<.001	.18	<.001
**Online health information** **seeking** **(mean 1.03, SD 0.99)**								
	*r*	0.15	0.16		0.18	1	−0.01	−0.08
	*P* value	<.001	<.001		<.001	—	.63	<.001
**Cancer information avoidance** **(mean 2.76, SD 0.98)**								
	*r*	0.05	0.11		0.03	−0.01	1	0.27
	*P* value	.01	<.001		.18	.63	—	<.001
**Fatalistic beliefs about cancer prevention** **(mean 3.15, SD 0.80)**								
	*r*	0.12	0.15		0.15	−0.08	0.27	1
	*P* value	<.001	<.001		<.001	<.001	<.001	—
**Age** **(mean 33.98, SD 10.88 years)**								
	*r*	0.07	0.08		0.10	0.01	0.20	0.27
	*P* value	.001	<.001		<.001	.50	<.001	<.001
**Sex** **(female=1 and male=0)**								
	*r*	0.01	0.04		0.08	0.07	0.04	0.08
	*P* value	.76	.07		<.001	.006	.05	<.001
**Education** **(mean 3.74, SD 1.13)**								
	*r*	−0.01	−0.01		−0.04	0.11	−0.10	−0.19
	*P* value	.59	.78		.05	<.001	<.001	<.001
**Personal income** **(** **mean 5.51, SD 1.94)**								
	*r*	0.01	0.03		0.01	0.01	0.13	0.11
	*P* value	.63	.22		.77	.94	<.001	<.001
**Health status** **(mean 3.98, SD 0.76)**								
	*r*	−0.33	−0.29		−0.08	−0.10	−0.06	−0.05
	*P* value	<.001	<.001		<.001	<.001	.002	.02
**Chronic disease** **(yes=1 and no=0)**								
	*r*	0.12	0.12		0.04	0.07	0.08	0.12
	*P* value	<.001	<.001		.04	.001	<.001	<.001
**Family cancer history** **(yes=1 and no=0)**								
	*r*	0.14	0.15		0.12	0.06	0.05	0.10
	*P* value	<.001	<.001		<.001	.001	.02	<.001
**Health insurance** **(yes=1 and no=0)**								
	*r*	−0.01	0.03		0.03	0.03	0.01	0.06
	*P* value	.49	.19		.18	.23	.92	.001

^a^Not applicable.

### Mediation and Moderated Mediation

To assess the mediating effect, the bias-corrected bootstrapping method was used to estimate the path CI. The results of bootstrapped CIs, with 5000 resamples, showed that cancer risk perception indirectly reduced cancer fatalism through OHIS (95% CI –0.010 to –0.003) but not through CIA (95% CI –0.014 to 0.003). In addition, the results supported an indirect effect of cancer worry on cancer fatalism, as mediated by OHIS (95% CI –0.012 to –0.004]) and CIA (95% CI 0.009 to 0.025). Hence, hypothesis 7 and hypothesis 8b were supported but not hypothesis 8a.

**Figure 3:**
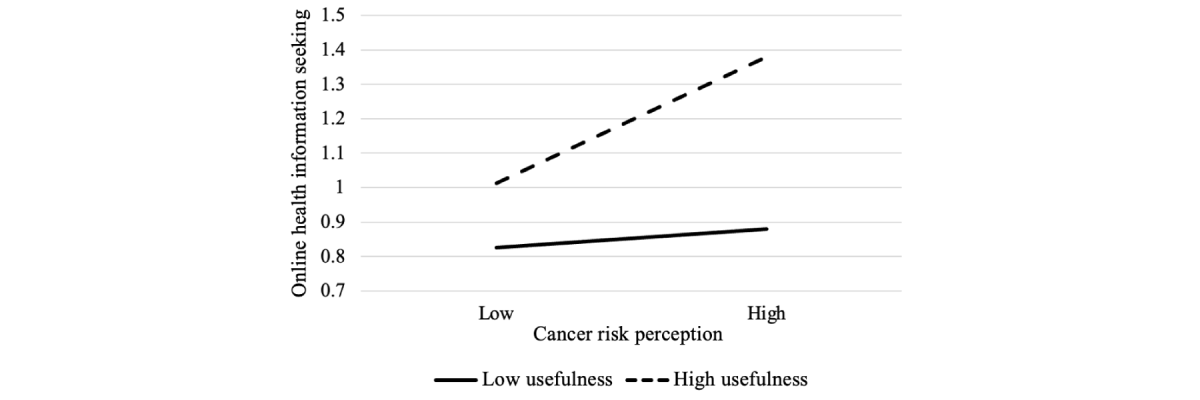
Moderating effect of perceived usefulness of online cancer information on the relationship between cancer risk perception and online health information seeking; the higher value is 1 SD above the mean, and the lower value is 1 SD below the mean.

As for the moderating effects, the results revealed that there was a main effect of the perceived usefulness of online cancer information on OHIS (β=.17; *P*<.001) but not for CIA (β=−.01; *P*=.58). More importantly, there was a significant interaction effect between cancer risk perception and perceived usefulness in predicting OHIS (β=.06; *P*=.01). The results revealed that the simple slope of the relationship between cancer risk perception and OHIS differed significantly from 0 when the perceived usefulness of cancer information was 1 SD above the mean (β=0.14; SE=0.05; *P*<.001) but not 1 SD below (β=0.06; SE=0.04; *P*=.11). This indicates that the positive association between cancer risk perception and OHIS was salient only among participants who perceived online cancer information to be of high usefulness but not among those who deemed the information was of low usefulness (Figure 3). However, there was no significant interaction effect between cancer risk perception and perceived usefulness in predicting CIA (β=−.04; *P*=.18).

**Figure 4:**
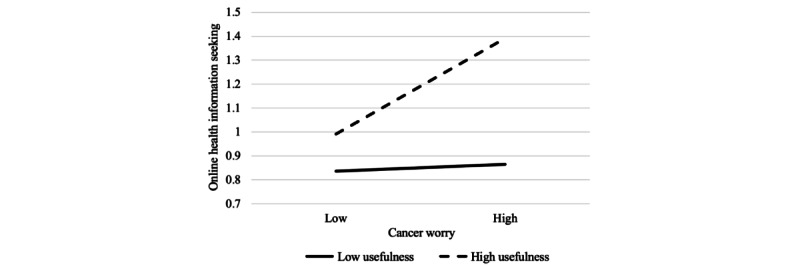
Moderating effect of perceived usefulness of online cancer information on the relationship between cancer worry and online health information seeking; the higher value is 1 SD above the mean, and the lower value is 1 SD below the mean.

Moreover, a significant interaction between cancer worry and information usefulness was observed in predicting OHIS (β=.10; *P*<.001). The results of the simple slopes revealed that when online cancer information was perceived as of high usefulness, worried participants frequently acquired health information on the web (B=0.18; SE=0.04; *P*<.001). However, this conditional effect of cancer worry was not observed when online cancer information was perceived as of low usefulness (B=0.02; SE=0.04; *P*=.61; Figure 4).

**Figure 5:**
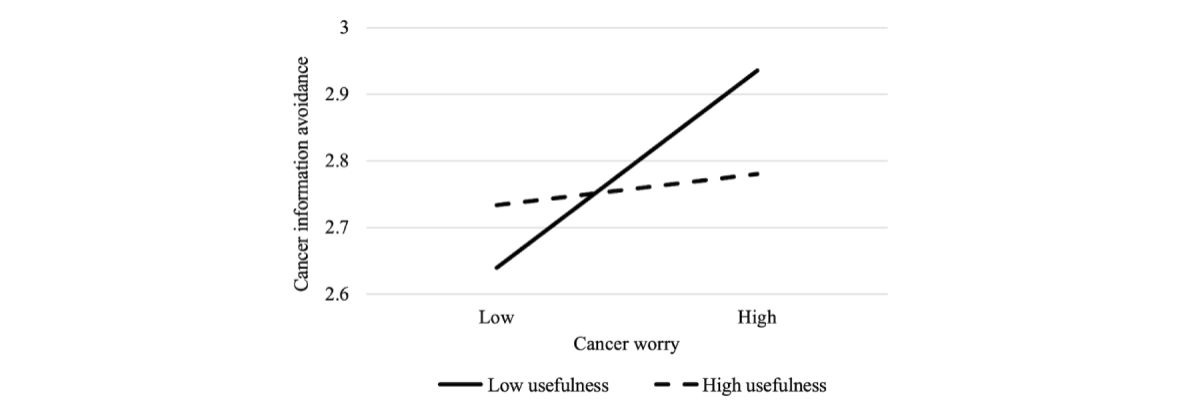
Moderating effect of perceived usefulness of online cancer information on the relationship between cancer worry and cancer information avoidance; the higher value is 1 SD above the mean, and the lower value is 1 SD below the mean.

Furthermore, a significant interaction between cancer worry and perceived usefulness was detected in predicting CIA (β=−.07; *P*=.009). Specifically, the positive association between cancer worry and CIA existed only for people who rated the usefulness of online cancer information as low (B=0.16; SE=0.04; *P*<.001) but not for those who scored the usefulness as high (B=0.06; SE=0.04; *P*=.09; Figure 5). In sum, hypotheses 9a, 9c, and 9d were supported but not hypothesis 9b.

The results also displayed significant moderated mediation effects (Table 2). The perceived high usefulness of cancer information strengthened the indirect negative influence of cancer risk perception and cancer worry on cancer fatalism through OHIS. However, a lower level of perceived usefulness significantly intensified the indirect positive influence of cancer worry on cancer fatalism through CIA.

**Table 2:** Conditional indirect effects of cancer risk perception and cancer worry on cancer fatalism through online health information seeking (OHIS) and cancer information avoidance (CIA) at different levels of perceived cancer information usefulness. B represents the unstandardized coefficient; italicized values indicate significant effects.

Independent variables (cancer risk perception and cancer worry), mediators (OHIS and CIA), and moderator (low, mean, and high perceptions of cancer information usefulness)				Dependent variable: cancer fatalism				
				B		SE		Bootstrap, 95% CI
**Cancer risk perception**								
	**OHIS**							
		Low (mean–1 SD)	–0.003		0.002		–0.008 to 0.000	
		Mean	–*0.005*		0.003		–*0.012* to *–0.001*	
		High (mean+1 SD)	–*0.010*		0.003		–*0.018* to *–0.002*	
	**CIA**							
		Low (mean–1 SD)	0.011		0.006		–0.002 to 0.023	
		Mean	0.009		0.005		–0.001 to 0.019	
		High (mean+1 SD)	–0.004		0.007		–0.017 to 0.013	
**Cancer worry**								
	**OHIS**							
		Low (mean–1 SD)	–0.002		0.002		–0.005 to 0.002	
		Mean	–*0.007*		0.003		–*0.014* to *–0.001*	
		High (mean+1 SD)	–*0.011*		0.004		–*0.019* to *–0.004*	
	**CIA**							
		Low (mean–1 SD)	*0.035*		0.007		*0.022* to *0.047*	
		Mean	*0.021*		0.005		*0.011* to *0.030*	
		High (mean+1 SD)	0.010		0.006		0.000 to 0.024	

## Discussion

### Principal Findings

This study reveals a dual-mediation pathway linking distinct cancer mental conditions to cancer fatalism, focusing on different information behaviors and considering the moderating role of the perceived usefulness of online cancer information. Findings from the HINTS-China data revealed that cancer risk perception and cancer worry were positively associated with OHIS. Consistent with previous studies [28,30], individuals who perceived a high susceptibility to getting cancer and felt worried about it tended to actively engage in OHIS, such as looking for exercise, weight control, or fitness information and exchanging health-related information on the internet. As such, the results suggest that both affective-cognitive (cancer worry) and cognitive (cancer risk perception) mental cognitions can serve as driving forces for people’s self-protective behaviors, such as health information acquisition.

However, the results indicated a different relationship between cancer worry and CIA when compared to risk perception and CIA, such that cancer worry rather than risk perception was positively associated with CIA. This finding might suggest that unlike risk perception, which has been widely noted as a problem-solving mechanism that leads to active information seeking [13,31], cancer worry tends to increase both general health information seeking and cancer-specific information avoidance, with mixed findings in the literature [58-60]. On the one hand, the finding that cancer worry was positively associated with both OHIS and CIA suggests the operation of moderating factors (eg, message characteristics) that facilitate seeking behaviors in some circumstances but avoidance actions in other contexts. On the other hand, psychologically, cancer worry is closely related to negative emotions, such as fear and anxiety. As noted by uncertainty management theory, information avoidance serves as a way of managing uncertainty and providing an escape from negative emotions [61]. This avoidance behavior tends to be more pronounced when confronting threatening and complex cancer information that may bring about more confusion and mental discomfort, even though it might compromise treatment. Hence, the results highlight that cancer risk perception and cancer worry should not be treated as homogeneous entities or used interchangeably because of their varying functions and effects.

Our study also found that OHIS was negatively associated with cancer fatalism, while CIA was positively related to it. In accord with previous studies [8,41], health information seeking, particularly via the internet, offers people diverse formats and depths of information across various health topics, helps specify a diagnosis or treatment plan, and provides clarity about prognoses. All these outcomes contribute to individuals’ increased health literacy and cancer knowledge, which are critical in reducing individuals’ negative coping needs embedded in cancer fatalism. In addition, OHIS offers people more opportunities to interact with others in social media communities and support groups, providing a broad sense and social proof that many others are active in engaging in self-protective behaviors for cancer prevention [43]. These perceptions help reduce people’s cancer fatalism, especially in societies that tend toward collectivism, such as China. In contrast to this study’s findings about OHIS, the finding of a positive association between CIA and cancer fatalism implies a detrimental influence of CIA on cancer prevention. Consistent with previous studies [10], people who refused to be exposed to cancer information delayed the discovery of positive information, thus maintaining their biased perceptions of their actual risks and self-agency. This biased belief is closely related to individuals’ tendencies to avoid physicians, other forms of help, and preventive screening [60,62]. These behaviors exacerbate individuals’ health risks, especially for those who are vulnerable to cancer for whom early detection is quite literally a life-or-death matter.

Another key finding pertains to the moderation effect. The results indicate that people only sought health information on the internet when they perceived it to be useful. If they deemed information to be useless, they tended to avoid it despite their cancer worries. Such results confirm the central postulate of the updated EPPM—the additive model—which suggests that higher levels of each threat and efficacy would lead to a more favorable impact, and the interaction effects between the threat and efficacy are additive [34]. In addition, the mediating effect of people’s information behavior on cancer fatalism was found to be contingent upon perceived information usefulness. This finding is consistent with the 3-stage model developed by Street [63], which highlights the vital role that positive experiences play in producing desired health outcomes out of user-media-message interactions. Particularly in the context of China, researchers have long questioned the problematic digital information environment and expressed concern about the negative influences of poor-quality health information, which are exacerbated by low levels of health literacy [64]. Therefore, positive media message characteristics (eg, information usefulness) are particularly important to encourage people to engage with more adaptive information behavior to better reap health benefits and combat cancers. Useless and low-quality cancer information may make people frustrated and overwhelmed, dampening their information seeking and even spurring CIA that leads to cancer fatalism. Hence, the results reinforce a challenging but imperative public health goal of providing more useful, understandable, and high-quality cancer information for people in China, especially in the digital era.

### Theoretical and Practical Implications

This study has contributed new insights to inform future research on health-related information behavior and the EPPM. First, in contrast to some previous studies that primarily focused either on information seeking behavior or information avoidance, a strength of this study is that it considers both information-related behaviors, which are of equal importance in understanding the development of fatalistic beliefs about cancer prevention. More to the point, this study broadens the scope of the EPPM by incorporating cancer fatalism, which reflects individuals’ negative behavioral coping tendencies, as a fear control response, and exploring its connection with both OHIS and CIA. This expansion helps elucidate the differing implications of these 2 distinct appraisals on fatalistic beliefs concerning cancer prevention. Second, conceptualizing the perceived usefulness of online cancer information as one of the manifestations of response efficacy, this study adds a new perspective to the EPPM and the literature on health-related information management. Third, building upon the cancer-related mental condition model [14], this study has taken a step further to investigate how distinct cancer mental conditions influence disparate information behavior differently, which contributes to the theoretical advancement of the effects of cancer-related affective responses and cognitive thoughts on cancer communication. In addition, Witte [65] has demonstrated that the “danger control processes are primarily cognitive processes,” whereas the “fear control processes are mainly emotional processes.” By establishing the positive relationship between cancer worry (an affective-cognitive condition) and OHIS (a danger control process) as well as the positive relationship between cancer worry and CIA (a fear control process), this study contributes to the EPPM by highlighting the dual nature of cancer worry in engaging the 2 different appraisals proposed by the EPPM. This paves the way for future EPPM research to thoroughly explore how various cancer-related mental conditions (eg, affective, cognitive, and affective-cognitive) may either motivate or inhibit individuals in safeguarding themselves against threats such as cancer as well as by which conditions. This is particularly significant as the EPPM has traditionally focused on purely emotional appeals (eg, fear) or cognitions (eg, perceived susceptibility and perceived severity).

The findings also provide useful implications for cancer communication and control. First, cancer worry has both positive and negative influences in our model; thus, developers of future health campaigns aimed at increasing people’s risk perceptions should be cautious about unintended outcomes. They must be vigilant in avoiding negative affective responses toward cancer threats to avoid eliciting excessive cancer worry that provokes an “ostrich effect.” More tailored communication strategies are needed to promote rational thinking about cancer and personal risk, avoid inflating anxiety, and avert CIA and possible anxiety disorders [58]. Second, in clinical settings, it would be useful for physicians to identify and pay special attention to patients with high trait anxiety. Practitioners should help them attenuate unnecessary worries and anxiety through affectionate and personalized counseling. Instead of information avoidance, training in new coping skills (eg, breathing exercises, relaxation strategies, and mental imagery exercises) should be incorporated into health education and counseling. Given the moderating role of perceived information usefulness and the effectiveness of OHIS in reducing cancer fatalism, health educators are encouraged to disseminate useful, accurate, and feasible information with concrete skill sets that are easy and effective in fighting cancer threats. Furthermore, considering the potential of OHIS to alleviate cancer fatalism, public health practitioners need to make efforts to promote information seeking behavior that informs and empowers people, particularly for certain groups who are vulnerable to cancer fatalism (eg, those with low educational attainment or low health literacy).

### Limitations and Suggestions for Future Research

Several limitations are worth examining more closely in future research. First, the use of cross-sectional data precluded the causal inferences in this study. To determine causality, longitudinal studies with panel data are encouraged to affirm the temporal order. Second, due to the use of secondary data, cancer worry, information usefulness, and CIA were assessed with single items. Although these measures have been frequently used in previous studies [28,54,55], future research would ideally use multiple-item scales to enhance content validity. Third, this study did not directly investigate what types of external stimuli can trigger individuals’ threats but only examined the relationship between individuals’ perceived cancer threats and subsequent coping responses. To expand upon the EPPM, it is essential for future research to use experimental methods to evaluate the message characteristics that can effectively induce adequate levels of risk perceptions, thereby encouraging adaptive actions. This endeavor holds significant promise for advancing our theoretical understanding of how various persuasive messages, including those designed to induce fear, are processed within the theoretical framework of the EPPM.

Moreover, given the specific scope of this study, our research model exclusively examined the relationship between 2 appraisals and a fear control outcome (ie, cancer fatalism), without delving into potential danger control outcomes, such as changes in belief, attitude, and behaviors concerning cancer prevention. Future research is encouraged to incorporate potential manifestations of danger control in providing a more comprehensive understanding of both fear control and danger control outcomes and their relationship with appraisals of threat and efficacy. Finally, cancer fatalism is a multidimensional construct that has been conceptualized differently across the cancer continuum [5]. A direct extension of this study would be to include other aspects of fatalism, such as fatalistic beliefs about cancer treatments among survivors of cancer who are receiving treatments.

### Conclusions

Cancer is a threatening health problem and becoming an increasing burden on a global scale. Although a great proportion of cancer cases can be prevented and cured, cancer fatalism is one of the major obstacles for cancer prevention and control. This study provides evidence that OHIS is an effective mechanism for reducing cancer fatalism and minimizing CIA is necessary to allay fatalistic beliefs about cancer prevention. To facilitate healthy behavior, apart from disseminating more useful cancer information that assists people in coping with cancer threats, future endeavors should heighten rational risk perception while being cautious about elevating unnecessary cancer worry that may prompt information avoidance.

## Abbreviations

CIA: cancer information avoidance
EPPM: extended parallel process model
HINTS: Health Information National Trends Survey
IRB: institutional review board
OHIS: online health information seeking
SRMR: standardized root mean square residual

## Data Availability

The data sets generated or analyzed during this study are available from the corresponding author upon reasonable request.
